# Reevaluating the protective effect of smoking on preeclampsia risk through the lens of bias

**DOI:** 10.1038/s41371-023-00827-9

**Published:** 2023-04-11

**Authors:** Merida Rodriguez-Lopez, Maria Fernanda Escobar, Juan Merlo, Jay S. Kaufman

**Affiliations:** 1 Faculty of Health Science, Universidad Icesi, Cali, Colombia; 2 Unit for Social Epidemiology, Faculty of Medicine, Lund University, Malmö, Sweden; 3 Clinical Research Center, Fundación Valle del Lili, Cali, Colombia; 4 Department of Obstetrics and Gynecology, Fundación Valle del Lili, Cali, Colombia; 5 Department of Epidemiology, Biostatistics, and Occupational Health, McGill University, Montréal, QC Canada

**Keywords:** Risk factors, Medical research

## Abstract

Preeclampsia is a hypertensive disorder that is usually diagnosed after 20 weeks’ gestation. Despite the deleterious effect of smoking on cardiovascular disease, it has been frequently reported that smoking has a protective effect on preeclampsia risk and biological explanations have been proposed. However, in this manuscript, we present multiple sources of bias that could explain this association. First, key concepts in epidemiology are reviewed: confounder, collider, and mediator. Then, we describe how eligibility criteria, losses of women potentially at risk, misclassification, or performing incorrect adjustments can create bias. We provide examples to show that strategies to control for confounders may fail when they are applied to variables that are not confounders. Finally, we outline potential approaches to manage this controversial effect. We conclude that there is probably no single epidemiological explanation for this counterintuitive association.

## The puzzling association and its biological explanation

Preeclampsia (PE) is conceptualized as a hypertensive disorder that affects human pregnancy after 20 weeks of gestational age (GA) [1]. It is a public health problem that increases the risk of maternal morbidity and mortality and relates to long-term cardiovascular diseases [2]. Most cases become clinically evident during the third trimester [3]. The cumulative incidence of PE increases from 0.44% between the 20 and 25th week to 3.8% in the 41st week of gestation [3]. Proteinuria together with a rise in blood pressure (BP) and signs/symptoms of organ and uteroplacental dysfunction are features of the disease’s clinical definition. More recently, PE is diagnosed even without proteinuria [4]. For many years this multisystem disorder was thought to be caused by placental insufficiency; however, recent studies have demonstrated that cardiac remodeling can be present even before the onset of PE [5], suggesting a cardiovascular origin for PE [6].

Many observational [7–10] and systematic reviews [11–13] have pointed to a protective effect of smoking in PE risk. This puzzling association has been reported for gestational hypertension (GH) (OR 0.74, 95% CI 0.69–0.79) and PE (OR 0.65, 95% CI 0.58–0.73) [13], for term or preterm PE, for smoking before [14] or during pregnancy [15], and for cohort (RR 0.68, 95% CI 0.67–0.69) and case-control studies (OR 0.68, 95% CI 0.57–0.81) [16]. In contrast, other studies have shown that smoking during pregnancy increases the risk of PE [17], increases the risk just among taller women [18] or increases both systolic and diastolic BP with no association with PE [19]. This protective effect of tobacco on PE risk contrasts with its deleterious effect on severe hypertension and cardiovascular diseases in the population [20, 21]. PE and chronic hypertension (CH), share similar risk factor profiles except for smoking. For these reasons, this association is still a source of debate.

Arguments in favor of the causal effect of smoking on PE highlight the role of nitric oxide, a potent vasodilator [22]. However, smokers have decreased levels of circulating nitric oxide metabolites [12]. It has been proposed that combustion products in tobacco, including carbon monoxide but not constituents of tobacco, such as nicotine, are responsible for the protective effect [15]. Carbon monoxide mimics nitric oxide and therefore might replicate its effects, inhibiting placental apoptosis, necrosis, and the production of antiangiogenic proteins such as sFlt1 [22]. However, higher levels of blood carboxyhemoglobin, a stable complex of carbon monoxide and hemoglobin, are associated with a higher risk of PE. Animal models have also pointed to an anti-inflammatory effect of nicotine, which might attenuate its capacity to increase systemic BP [23].

Unexpected associations between smoking and health outcomes have been previously reported in the field of perinatal epidemiology [24, 25]. For example, smoking is a risk factor for low birth weight and mortality. However, among those born with low weight, smoking appears to work as a protective factor for mortality [24]. These apparent paradoxes have been explained by selection bias [26]. Recently, some papers have addressed the protective effect of smoking on PE considering possible sources of selection bias one at a time [27, 28]. In this manuscript we suggest multiple mechanisms for bias without going deeper into their statistical structure. We first review key concepts in epidemiology: confounders, mediators, and colliders. Then we discuss the potential impact of selection bias due to exclusion, losses of women and pregnancies, misclassification, and covariate over-adjustment [29]. Finally, we outline some potential strategies to better understand this puzzling association.

## Key definitions in epidemiology

Most readers are familiar with the definition of a confounder; however, the concepts of colliders and mediators may be less frequently used among clinical researchers. A “confounder” is commonly understood as a variable that meets three conditions: (1) it is an independent risk factor for the outcome; (2) it is related to the exposure without being affected by the exposure and consequently, (3) it does not lie on the pathway between exposure and the outcome [30]. More generally, a confounder is a common cause of the exposure and the outcome, or a proxy for such a cause [31]. The definition of confounding in a particular exposure-outcome association relies on the structure of the relationships between variables in each causal framework [32]. Well-known strategies to control confounding include but are not limited to restriction, matching, stratification, adjustment, propensity scores, and randomization. In some cases, a variable appears to meet the three conditions listed above but does not generate confounding because, rather than being a common cause, it is a common effect of the exposure and the outcome.

One specific common effect is known as a “collider” [26]. In general, a collider is a third variable that is influenced by both the exposure and the outcome (or by a cause of the exposure and a cause of the outcome). For example, PE and smoking can both lead to renal diseases, then renal disease is a common effect of both PE and smoking. Contrary to confounders, if the collider is controlled for by design or analysis, it can induce a spurious association between the exposure and the outcome which is known as collider bias [33]. A more detailed definition of collider can be found elsewhere [34, 35]. Moreover, renal disease can cause PE, but, as it cannot cause smoking, then it is not a confounder unless a diagnosis influences a smoker to quit. In some causal frameworks, smoking could cause renal disease and then renal disease could cause PE. If this were the case, renal disease can be considered a “mediator” of the effect of smoking on PE. A mediator is a consequence of the exposure and a subsequent cause of the outcome. That is, a variable that lies on the pathway between exposure and outcome. Strategies to reduce confounding bias might also fail if they are applied to mediators without additional assumptions, as it is discussed below.

**Fig. 1:**
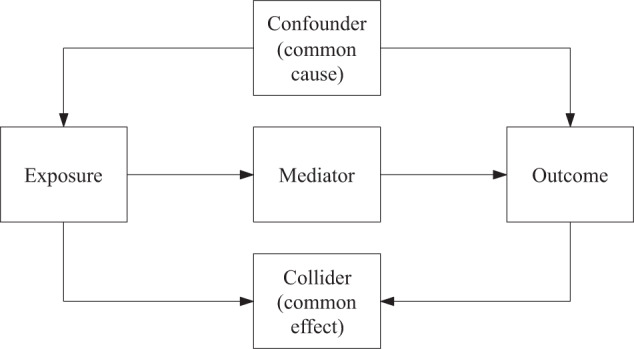
Basic structure of the relationships between variables within a causal framework perspective. Arrows indicate the direction of causality. Confounder, mediator, and collider refer to the exposure-outcome association.

Figure 1 illustrates the roles of variables and the ways in which they are causally connected [26, 36]. One graphical representation of these roles is known as a directed acyclic graph (DAG), which is a valuable tool for causal inference [31]. In DAGs, the distinction between confounders, mediators, and colliders is made explicit, such as (1) we might want to separate the direct and indirect effects (the effects through the mediator) of an exposure, and (2) controlling for confounders can reduce bias, but controlling for a collider can increase bias [36]. In terms of formal DAG rules, stratification and conditioning on a confounder block but performing such strategies on a collider unblocks a “backdoor path” between the exposure and the outcome [26]. Up to date, there are other statistical approaches based on standardization (weighting), g-methods and doubly robust methods [37], rather than conditioning, that enable accounting for colliders without generating collider stratification bias.

## The epidemiological view of the puzzling association

### Eligibility criteria and losses to follow-up of at-risk women

**Fig. 2:**
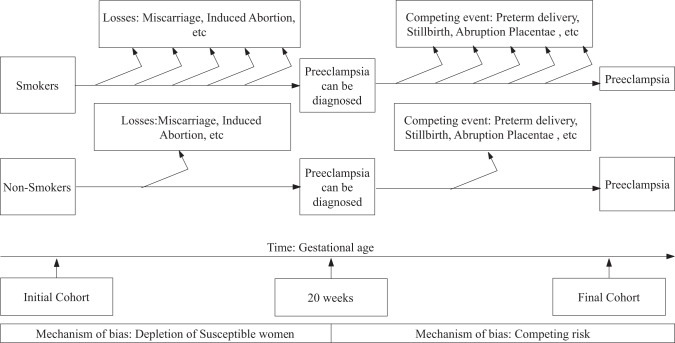
Potential losses of women at risk during pregnancy. The number of arrows moving out from horizontal lines represent the loss of follow-up among smokers and non-smokers. Left- (below 20 weeks of gestational age) and right-hand sides (above 20 weeks of gestational age) draw two potential mechanisms of bias: the left side shows that losses before 20 weeks create a study cohort that is depleted of cases susceptible to the effect of smoking, while the right side shows that competing events, which are known to be more frequent among smokers, preclude the incidence of PE in that subgroup.

PE is generally diagnosed after 20 weeks of GA. However, there are rare PE cases diagnosed before 20 weeks, mostly associated with molar pregnancy, triploid or antiphospholipid syndrome. Recently, some PE cases have been identified even in the absence of such pathologies [38]. A high BP value <20 weeks is most likely diagnosed as CH [39]. Consequently, the follow-up of women usually starts at 20 weeks of GA in cohort studies, and when using prevalent cases as in case-control and birth-registered studies, those women <20 weeks are excluded from the analysis [10, 40, 41]. Under the current clinical and epidemiological definition of PE, it can only present itself among those pregnancies that continue beyond 20 weeks of gestation. Therefore, there is a time frame within which, even if there is smoke exposure, PE cannot be diagnosed. The restriction of the study population to those pregnancies >20 weeks create a truncated cohort as depicted in Fig. 2 (left side) that can generate bias if smoking is associated with the cause of losses of at-risk women.

Pre-pregnancy smoking doubles the risk of early pregnancy loss [42, 43], including miscarriage [44], with most cases occurring before 14 weeks [45]. Overall, 10–20% of clinically recognized pregnancies will end in early pregnancy loss [46]. The true incidence of miscarriage may increase to 30% [47], as many miscarriages occur before a mother realizes she is pregnant. In fact, the incidence ranges from 18 to 62% when using human chorionic gonadotropin for pregnancy detection [48]. It has also been reported that up to 78% of fertilized ova may be lost. Therefore, the number of conceptions is much higher than the number of detected pregnancies, which is also higher than the number of deliveries. Early pregnancy loss produces a subpopulation cohort that is depleted of those susceptible to the smoking effect at the time by which PE is diagnosed, which might create selection bias at baseline [49]. This phenomenon was demonstrated using a simulation study by Lisonkova and Joseph [27]; however, it did not fully explain the inverse and counterintuitive findings [50].

### Competing events

Smoking also increases the risk of adverse perinatal outcomes during the second and third trimesters, which also prevents the incidence of PE by ending the pregnancy. Smoking during pregnancy has been associated with stillbirths after 22 weeks [51], and a doubling of the risk of a premature birth before 34 weeks [52], placental abruption, and fetal growth restriction. These and other conditions lead to an increased likelihood of induced labors and elective cesarean delivery [53]. They are then considered competing events, as shown in Fig. 2 (right-hand side), because their occurrence precludes the onset of PE. During the follow-up of a pregnant woman, the end of pregnancy can be due to a healthy birth, a birth due to PE, or a birth resulting from another complication such as fetal distress, fetal growth restriction, or placental abruption. These complications before the onset of PE impede its occurrence because they usually lead to medical intervention and iatrogenic preterm births [53]. They causally ‘compete’ with PE for the pregnancy’s end.

**Fig. 3:**
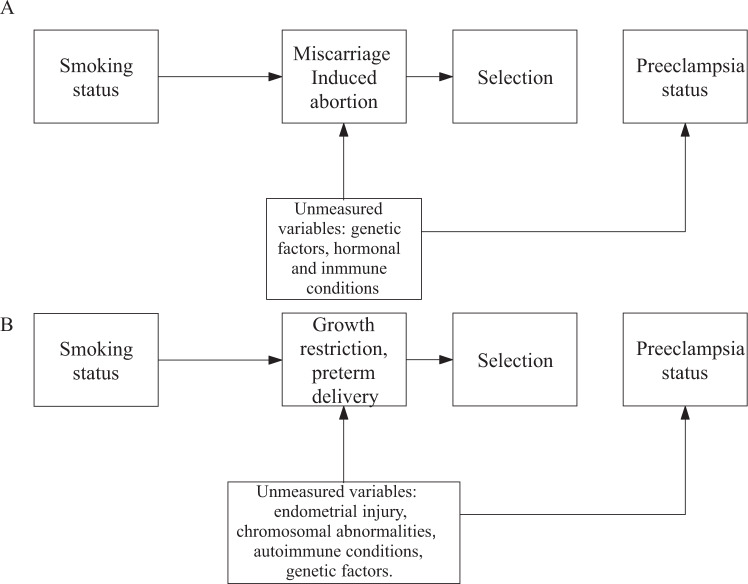
Collider bias occurs when the selection of the study population is restricted to pregnancies which survive the effect of tobacco. **A** During the first trimester (**B**) During the third Trimester. Arrows indicate the direction of causality. Gray line represents the association of interest.

The competing event could be considered a collider: it is caused by smoking and share common (and often unmeasured) causes with PE. Figure 3 represents potential unmeasured confounders in the relation between the collider and PE before (A) and after (B) week 20. If it is not possible to control such relevant unmeasured factors, they collide with smoking in the competing event, and the path between smoking and PE is open according to DAG rules. If unmeasured variables were measured, they could be included in the statistical model as confounders of the relation between the collider and the outcome, and then the bias could, in principle, be controlled. All these scenarios highlight the importance of following the entire cohort of women throughout their pregnancy, rather than only those who give birth, and to account for the reasons and characteristics of women who exit pregnancy at various times.

The loss of women at-risk affects the likelihood of an individual being sampled, and therefore affects selection. This is the case for studies where the population is selected on birth register information or retrospective studies which use prevalent cases [37]. Losses before, and after 20 weeks of GA need to be carefully considered in cohort studies as most traditional survival approaches require non-informative censoring, that is, censored women have the same probability of experiencing PE than those remaining in the study, which is not the case here.

Likewise, GH is defined as high BP after week 20, in the absence of proteinuria, organ and uteroplacental dysfunction. Despite it can be considered a milder form of PE, GH is usually considered as a different condition [1]. The incidence of GH versus PE increases earlier during pregnancy. As matter of fact, 50% of GH cases occur before the 35th week of GA [3]. Once GH is diagnosed, closer follow up visits, pharmacological treatment and, in some cases, labor induction before week 37 are offered to women. Hence, the prompt identification and treatment of GH early in pregnancy might impede the progression from GH to PE. Therefore, GH could be either a less severe form of PE or a competing event in the relation between smoking and PE.

### Confounders

Temporal relations between variables and the period of observation are essential in identifying the role of a variable within a causal framework, to then determine whether to apply confounding control strategies [32]. There are controversial findings regarding smoking being a risk factor for CH [54–56]. If smoking increases the risk of masked hypertension, [57], and the day-time BP by up to 5–6 mmHg when smoking two cigarettes per hour [58], it could increase the likelihood of incident hypertension [56] and the chance that a patient can be diagnosed with hypertension. Once CH is present, smokers are advised to quit, especially those planning to get pregnant.

**Fig. 4:**
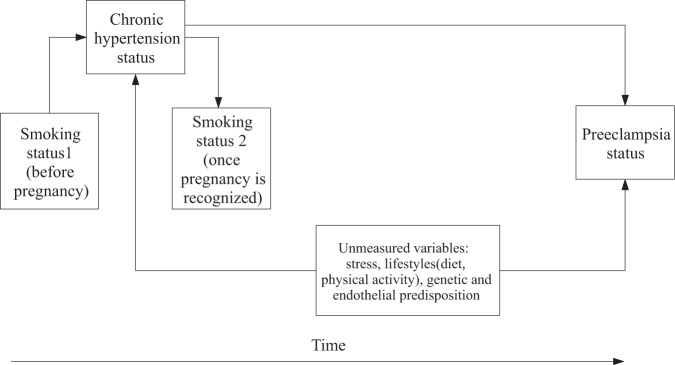
Collider bias occurs when adjusting the analysis for a variable that is a common effect both of the exposure and of a cause of the outcome. Arrows indicate the direction of causality. Gray line represents the association of interest.

In Fig. 4, CH can work as a mediator in the relationship between smoking at time 1 and PE, this would be the case of a woman that starts smoking in adolescence, after which is diagnosed with CH and after that becomes pregnant and develop PE. If CH is considered a mediator, one can assess the direct effect of smoking on the risk of PE, i.e., the effect that is not mediated through the effect of smoking on CH. The direct effect of smoking would be correctly estimated if: (1) there is no interaction between smoking and CH; that is, the effect of smoking on PE risk is the same in both CH + and CH- patients, and (2) the common causes (confounders) of the relation between the mediator (CH) and the outcome (PE) are controlled [59]. If common causes of both CH and PE are not controlled, the association between the mediator and the outcome is confounded and the mediator becomes a collider. Adjusting for a collider could create a biased association, as previously explained. CH is typically treated as a confounder using exclusion or another adjustment strategy [15, 60]. CH acts as a confounder in the relationship between smoking at time 2 and PE. That is, having a CH diagnosis subsequently affects both smoking status and the risk of PE. In such scenarios, the attempt to control for CH is reasonable.

All these temporal relations are usually unaccounted for in live-birth observational studies because the time of onset for each condition is not registered, and smoking is usually considered a time-fixed variable. Indeed, dependencies and temporal relationships between variables in a DAG are assumed to be the same for all women in the study population. Estimating direct effect and modeling all potential pathways is cumbersome due to data availability restrictions. In addition, birth registers do not usually include information about potential unmeasured confounders like lifestyle factors, which could also contribute to the counterintuitive association.

### Misclassifications

A source of misclassification bias is the inclusion of proteinuria for the diagnosis of PE, which is neither 100% sensitive nor specific. In addition, a young woman with previous unrecognized CH, might become normotensive beginning pregnancy, since BP usually drops during the first trimesters. Under current classification of hypertensive disorders in pregnancy, high BP values before 20th week of GA indicate the presence of CH, after which a new onset proteinuria or maternal organ dysfunction consistent with PE leads to diagnosis of PE superimposed on CH [1]. Persistent high BP beyond week 12 postpartum, in a woman with a previous diagnosed GH, indicates an unrecognized CH. Thus, any type of hypertension in pregnancy recorded at birth can be reclassified during postpartum period. Regarding the exposure, smoking status as a dichotomous variable, even if recorded at the beginning of pregnancy, ignores any cumulative effects from previous tobacco´s exposure. Information bias can be induced if the exposure is self-reported, if those who quit are treated as never exposed, or if those smoking even one cigarette in life are treated as smoker.

### The role of gestational age

GA has been used in stratified analysis. Adjustment for GA as a confounder or as a mediating variable will lead to bias when analyzing the association between prenatal factors and neonatal outcomes [61]. Under the smoking-PE causal hypothesis, GA is unlikely to create confounding as it is not a cause of smoking, unless smoking cessation increases with GA. However, the act of quitting smoking can only be present among smokers. One may argue that a certain GA is required to reach a diagnosis of PE according to current medical definitions, so that GA would be a necessary cause or a mediator. Luque-Fernandez analyzed GA as a mediator and reported an OR > 1 [28, 62]. Cases of postpartum PE diagnosed within 48 h and up to 6 weeks after birth were not included in that study. GA is the time frame from “conception”, or usually from the last menstrual period to a certain outcome (e.g., PE, delivery). In general, time window-at-risk to develop PE is equal to the duration of gestation. Therefore, GA can be treated as a time variable in survival analysis.

**Fig. 5:**
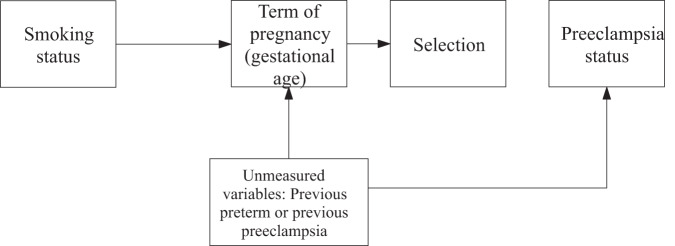
Collider bias occurs when stratifying or adjusting the analysis for a variable that is a common effect both of the exposure and of a cause of the outcome. Arrows indicate the direction of causality. Gray line represents the association of interest.

Dichotomized GA is more likely to be a collider, because it can be a common effect of smoking, and of an unmeasured cause of PE [63, 64] as shown in Fig. 5. Stratification on GA is implicit when clinical researchers define PE as preterm (<37 weeks) or term (≥37 weeks) [15], or as early-onset (<34 weeks) or late-onset (≥34 weeks) [65]. Preterm deliveries might preclude the incidence of PE at term. Early delivery due to other cause is then a competing event for most PE cases occurring at term. Maternal smoking and unmeasured confounders can be both associated with preterm delivery and PE [28]. This issue also applies when matching cases and controls by preterm/term delivery. Case-control designs are vulnerable to collider stratification bias, as selection into the study population is determined by the PE status. If smoking also influences the selection or the cause of the selection (e.g., GA), bias appears or is exacerbated [26].

## Unpacking the puzzling association

Similar “paradoxes” can appear in any medical field when the control for a confounder, mediator or a collider ignores the direction and temporal relations between variables. Some strategies to extricate the role of selection-bias in the presence of paradoxical effects are outlined below; however, further studies are warranted to evaluate the advantage of concurrent usage of these strategies.

1. To draw the causal diagram of the study question during the design, analysis and when interpreting the study results. The use of graphical tools such as DAGs [26] can help to identify sources of bias [66]. The temporal relationship between variables should be accounted for, as the role of a variable could change over time. In studies where the temporal relation between variables is not correctly accounted for, bias due to reverse causation must be acknowledged. Joint models including survival and longitudinal data simultaneously are now available in the field, however they do not allow for the application of different DAGs on different subsets of the study population and then modelling all pathways simultaneously.
2. Cohort studies starting before conception or very early in pregnancy and the use of the fetus at risk approach are preferred over studies based on birth registers. In general, paradoxical effects from observational studies, starting after the onset of exposure, should be interpreted with care [67–69].
3. To specify whether total, indirect, or direct causal effects are relevant to the research question. When assessing the direct or indirect effect of an exposure, a mediator may become a collider if there is unmeasured confounder between the mediator and the outcome. It is preferable to exclude the collider/mediator in the analysis if total effect is of interest.
4. To eliminate confounding, adjusting for a common cause is generally needed, while adjusting for a common effect is not. Inverse probability weighting and g-methods may be advantageous for addressing covariates without generating collider bias [70].
5. Dealing with GA is cumbersome in the analysis [61]. The use of the fetuses-at-risk approach [71], including GA as a time variable, and using a competing risk model [72, 73] are strategies to be considered for outcomes that occur before birth [74]. There are valid analytical approaches to manage mediators [75–77]. However, there is no consensus on the correct strategy to handle GA [78–80]. Sensitivity analysis to estimate the magnitude of the bias [81], and strategies to include mediators and competing events in survival analysis [82] might be necessary.

## Conclusion

“One size does not fit all” seems to apply when trying to explain the association between smoking and PE. Several sources of bias could explain this association including but not limited to eligibility criteria, early losses, competing events, the definition and misclassification of preeclampsia, inadequate adjustment, measurement errors of smoking, and unmeasured confounders. In addition, causal explanations based on studies where the temporal relation between variables is not guaranteed for each participant are challenging, since the same DAG´s structure and dynamics might not apply for the overall study population. More generally, estimating the average risk on a probabilistic approach for the determination of specific individual causal effects is frequently cumbersome [83]. To conclude, the reported biological protective effect of smoking on PE might be controverted by epidemiological reasoning.

## Data Availability

Data are contained within the paper.
